# Functional neural networks of honesty and dishonesty in children: Evidence from graph theory analysis

**DOI:** 10.1038/s41598-017-11754-4

**Published:** 2017-09-21

**Authors:** Xiao Pan Ding, Si Jia Wu, Jiangang Liu, Genyue Fu, Kang Lee

**Affiliations:** 1 Department of Psychology, National University of Singapore, Singapore, 117570 Singapore; 2 Dr. Eric Jackman Institute of Child Study, University of Toronto, Toronto, M5R 2X2 Canada; 3 School of Computer and Information Technology, Beijing Jiaotong University, Beijing, 100044 China; 4 Department of Psychology, Hangzhou Normal University, Hangzhou, 311121 China; 5 Department of Psychology, Zhejiang Normal University, Jinhua, 321004 China

## Abstract

The present study examined how different brain regions interact with each other during spontaneous honest vs. dishonest communication. More specifically, we took a complex network approach based on the graph-theory to analyze neural response data when children are spontaneously engaged in honest or dishonest acts. Fifty-nine right-handed children between 7 and 12 years of age participated in the study. They lied or told the truth out of their own volition. We found that lying decreased both the global and local efficiencies of children’s functional neural network. This finding, for the first time, suggests that lying disrupts the efficiency of children’s cortical network functioning. Further, it suggests that the graph theory based network analysis is a viable approach to study the neural development of deception.

## Introduction

Honesty is a highly valued virtue in all cultures of the world. However, people regularly lie in their daily lives^1^, and such deceit begins as early as two years of age^2^! Although extensive behavioral research has examined deception in children and adults for nearly a century^3,4^, only recently have researchers begun to examine the neural basis of deceptive behaviors. Most of the existing studies have only involved adults and have exclusively focused on identifying the focal brain areas related to dishonesty^5–13^. No study has taken a complex network approach to reveal how different brain regions interact with each other during honest vs. dishonest communication in children. The present study aimed to fill this major gap by examining the neural functional connectivity underlying children’s spontaneous honest and dishonest acts.

In the past decades, researchers have used various neuroimaging methodologies (fMRI, PET, tDCS, fNIRS) to investigate the neural correlates underlying honest and dishonest behavior^13–18^. Most of the existing neuroimaging studies have found that dishonest behaviors result in much broader brain activations than honest behaviors, and these activations mainly occur in the prefrontal cortex (PFC)^7,10,19,20^. Since the PFC plays a central role in cognitive control^21–23^, these findings support the idea that, compared to honest behavior, dishonest behavior is a more intensive cognitive control task^13,14,24,25^. To deceive, one must suppress the truth while they are lying. Cognitively, this act is highly demanding, because it requires a host of executive functions such as inhibition, working memory, and cognitive flexibility^15,19,26,27^. Also, activating and formulating a lie is more intentional and deliberate than activating the truth^25,28^.

To date, the existing studies have exclusively focused on identifying the focal brain areas related to honesty/dishonesty. However, dishonest and honest acts, like many other complex social-cognitive acts, never engage the brain in isolation. Rather, they inevitably involve dynamic interactions between different brain regions. In this study, we address this issue by comparing the differences of topological neural organizations of honest and dishonest acts. We took a complex network approach to analyze neural response data when children are engaged in honest or dishonest acts. We used the graph theory methodology^29–31^ to examine not only the differences in topological neural organizations between honest and dishonest acts, but also how such network topological differences develop with age. To measure children’s honest and dishonest behaviors, we used a novel behavioral paradigm we developed^5,32^. Unlike previous studies^33^ where experimenters asked children when and how to lie, this paradigm situated children in a naturalistic situation where they could decide to lie or tell the truth spontaneously out of their own volition. Furthermore, instead of comparing the truth-telling and lying behavior trial by trial, we divided the whole time course into two states (honest state vs. dishonest state) according to children’s first lie. By doing this, we aimed to examine how the brain network properties changed after a child told his/her first lie.

We tested two contrasting hypotheses concerning the neural correlates of honest and dishonest behaviors, especially focusing on the differences of network efficiency between the two. According to the “network enhancement” hypothesis, because deception is a cognitive control intensive task, dishonesty should engender greater neural network efficiency than honesty in the frontal-parietal system^34,35^, which is known to be actively involved in cognitive control^36,37^. That is, the neural network underlying dishonest behavior should become more effective at integrating information than that of honest behavior. In contrast, according to the “network disruption” hypothesis, dishonesty depletes cognitive resources and leads to impaired cognitive control^25,26,28^. Since the impaired cognitive control is associated with low efficiency in their neural network topology^34^, we hypothesized that dishonesty should lead to a greater reduction in neural network efficiency than honesty in the frontal-parietal system. Further, regarding developmental changes, previous studies have found that with increased age children become more apt at meeting cognitive-control challenges^38–40^.Thus, we hypothesized that the differences in the neural network topological properties between honesty and dishonesty would decrease with increased age.

## Method

### Participants

Fifty-nine right-handed children between 7 and 12 years of age (27 boys; *M*_age_ = 8.83; *SD* = 1.31) participated in the study. Parents or legal guardians first provided written informed consent prior to their children’s participation in the study. We then obtained oral assent from all child participants. The study was conducted in accordance with the NIH research ethics guidelines and approved by the Zhejiang Normal University research ethics committee.

## Procedure

Children were tested individually, seated in front of a computer screen, with an experimenter present in a quiet room. Before the game, children were told that a coin would appear on either the left or the right side of the screen and were instructed to guess on which side of the screen the coin would appear by moving their corresponding hand. Children put each of their hands in one of the two drawers of a desk so their hand movements would not be directly visible to the experimenter. After they made their guesses by moving one of their hands, the coin appeared on the right or left side of the screen. A message came up on the screen asking them whether the actual location of the coin was the same as their guess.

**Figure 1:**
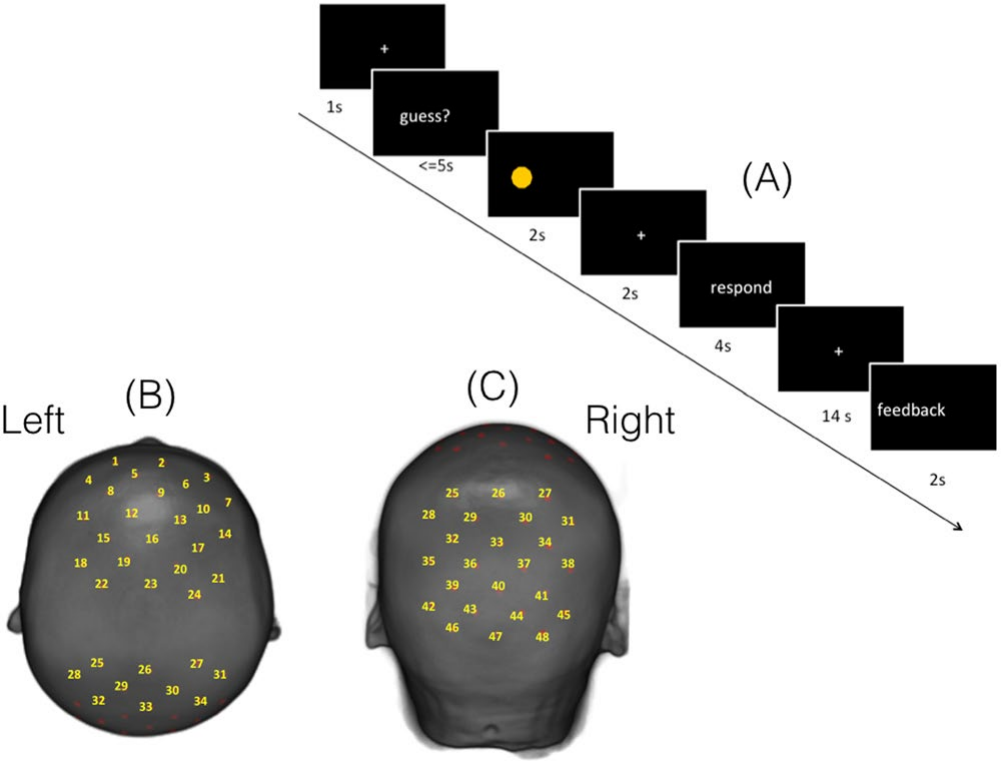
(**A**) An example of the guessing game. (**B**) The co-registration of 48 channels on Chinese children’s brain template (the frontal and parietal area). (**C**) The co-registration of 48 channels of brain template (the parietal and occipital area).

The children would receive or lose points according to their self-reported accuracy. Children were encouraged to try their best to obtain the highest score possible in order to receive their favorite gift. Children were made to believe that the experimenter would not know whether their self-reported accuracy was truly correct or incorrect. This procedure thus created a situation where children might be motivated to report falsely that their prediction was correct when in fact they predicted incorrectly. However, unbeknownst to children, we installed a hidden video recording system inside the drawers to record the movement of the children’s hands and the computer screen display simultaneously. Thus, whether children lied or told the truth about their accuracy could be verified by checking the experimenter’s recordings of children’s responses against the video recordings after the experiment was completed. E-prime 2.0 was used to present the stimulus. The whole experiment consisted of 36 trials (See Fig. 1A).

Upon completion of the experimental session, children were debriefed with their parents. Because the study involved minor deception, after the debriefing, they were given the opportunity to opt out of the study and have their data completely removed. All parents allowed their children’s data to be included in the study. The minor deception was necessary because it ensured that children would act naturally and decide to lie or tell the truth spontaneously out of their own volition.

## Data Acquisition

A 48 channels continuous wave system (ETG-4000, Hitachi Medical Co., Japan) was used in the present study. The optodes of the NIRS machine were fixed using two 9 × 9 cm^2^ rubber shells over the frontal and parietal areas. The shells were covered with a nylon-net to keep them attached to the head. The shells of 32 optodes, consisting of 4 × 4 arrays with eight light emitters and eight detectors, were capable of measuring the relative concentrations of hemoglobin at 48 points (See Fig. 1B&C). The lowest optodes were positioned along the Fp1-Fp2 line in accordance with the international 10-20 system for electroencephalography. The placements of the optodes in the dorsal bilateral frontal and parietal areas (because of the fixed optodes configuration, parts of occipital area were also included while testing the parietal area) were based on our hypotheses derived from the existing related studies^5,7,33,41,42^. The inter-optode distance was 30 mm, which allowed for measuring neural activities approximately 15–25 mm beneath the scalp. Optical data from individual channels were collected at 2 different wavelengths (695 and 830 nm) and analyzed using the modified Beer-Lambert Law for a highly scattering medium^43^. Changes in oxygenated hemoglobin ([oxy-Hb]) and deoxygenated hemoglobin ([deoxy-Hb]) signals were calculated in units of millimolar-millimeter (mM*mm)^44^. The sampling rate was set to 10 Hz.

## Data Analysis

**Figure 2:**
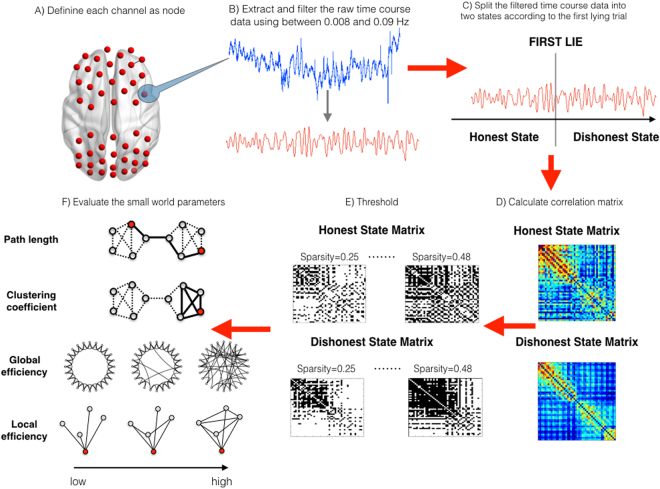
The steps for the functional brain networks analysis based on graph theory.

We only analyzed the [oxy-Hb] signals, because the [oxy-Hb] signals have high signal to noise ratios and are more sensitive to regional cerebral blood flow (rCBF) activity than the [deoxy-Hb] signals, which are noisy and unreliable^45–47^. We also did so in keeping with an existing fNIRS study that used the [oxy-Hb] signals to study cortical functional connectivity^48^. The steps for the functional brain analysis can be found on Fig. 2.

### Network construction

For individual data, we extracted the raw time course data from each channel and the raw data was filtered between 0.008 and 0.09 Hz to overcome drift and non-physiological noise^30^. We constructed the three pairs of matrices as follows:

First, to compare the differences of functional connectivity between the honest state and dishonest state in liars, we divided the time course data of each liar into two states using the time point of the first lie: “**the honest state**” was the time course data before the child told the first lie, and “**the dishonest state**” was the time course data after the first lie. Then we obtained the two 48 × 48 inter channel correlation matrices (the honest state matrix and the dishonest state matrix) for the two sessions of the time course data separately, by calculating the Pearson correlation coefficients of time courses between each pair of channels.

Second, to compare the differences of functional connectivity between liars and non-liars in the different states, we constructed two separate matrices for non-liars. Because the non-liars, by definition, never lied during the course of the experiment, to make the liar vs. non-liar comparison possible, we divided the raw time course data of each non-liar into two periods according to the average time points of ***the first lies told by the liars*** (11.5/36 of the whole time course data). These two periods for the non-liars are henceforth referred to as **the first and second periods**. Then, the two inter channel correlation matrices were computed for the two periods of time course data separately for the non-liars: the first period matrix, and the second period matrix.

Third, to examine whether the functional connectivity in the network of the liars and non-liars were fundamentally different, we constructed overall complex networks of the liars and non-liars regardless of the states or periods. Two inter channel correlation matrices were computed for the entire time series of each liar or non-liar: liar’s overall matrix, and non-liar’s overall matrix.

Next, a wide range sparsity threshold S was applied to all correlation matrices (*Sparsity* is defined as the number of actual edges in a network divided by the maximum possible number of edges in a network); its minimum was set so that the averaged node degrees of the network, to which the threshold was applied, was larger than 2 log(N), where N is the number of nodes^49^, and its maximum was set so that the network small-worldness scalar (σ) was larger than 1.1. This procedure generated a threshold range of 0.25 < *S* < 0.48 with an interval of 0.01. This threshold strategy produced networks that could estimate network properties with sparse properties and the minimum possible number of spurious edges.

### Network topological properties

For the brain networks at each sparsity level, five network parameters measures were computed: normalized clustering coefficient (γ) that measures network segregation, normalized characteristic path length (λ) that measures network integration, and the network small-worldness (σ)^50,51^, that measures the balance between network integration and segregation relative to comparable random networks. We also obtained two efficiency measures: global efficiency (E_glob_) and local efficiency (E_loc_)^52^. Global efficiency measures how easily information can be exchanged over the network, providing information on the communication efficiency of a network as a whole. Local efficiency reflects how well information can travel in the direct neighborhood of a node, and is often interpreted as the local information processing capacity of a network^31,52,53^.

The reason for choosing these five parameters to analyze is: First, the five network parameters measures (normalized clustering coefficient (γ), normalized characteristic path length (λ), the network small-worldness (σ), global efficiency (E_glob_) and local efficiency (E_loc_)) are the mostly commonly used properties in the graph-theory based analysis papers^54,55^. Second, the global and local efficiency were the most robust measure of brain network integrity^56^. Third, across the studies of functional networks, these five metrics had median intra-class correlations (ICCs) in the good or excellent ranges^57^. See Supplemental Methods for detailed information.

We calculated the area under the curve (AUC) for each network metric. The AUC for a general matrix was calculated over the sparsity range from S_1_ to S_n_ with an interval of ΔS, here S_1_ = 0.25, S_n_ = 0.48, and ΔS = 0.01. The AUCs provide a summarized scalar for the topological characterization of brain networks that is independent of a single threshold selection and sensitive to topological variations^54,55^.

### Statistics analysis

First, to compare the differences between the honest and dishonest states in liars, five repeated measures 2 (states: honest vs. dishonest) × 1 (age in years: continuous) ANOVAs were performed with states (honest or dishonest) as a within-subjects variable, participant’s age as a continuous variable, and the AUCs of each of the five network properties (γ, λ, σ, E_glob_ and E_loc_) as the dependent variables, respectively.

Second, to compare the differences between the first and second periods of non-liars, five repeated measures 2 (periods: first vs. second)×1 (age in years: continuous) ANOVAs were performed with periods (first or second) as a within-subjects variable, participant’s age as a continuous variable, and the AUCs of network properties (γ, λ, σ, E_glob_ and E_loc_) as the dependent variables, respectively.

Third, to compare whether there were fundamental differences between liars and non-liars, five 2 (types of participants: liars vs. non-liars) × 1 (age in years: continuous) ANOVAs were performed with types of participants (liars or non-liars) as between-subjects variable, participant’s age as a continuous variable, and the AUCs of each network property (γ, λ, σ, E_glob_ and E_loc_) as the dependent variables, respectively.

Fourth, to examine whether the liars’ network topological properties were associated with their lying behaviors, we conducted two separate analysis: 1) In order to examine whether liar’s overall network topological properties were associated with lying behavior, we conducted partial correlation analyses between the frequency of lying/FLT (the first lie trial) and the AUCs of each network topological property (γ,λ,σ, E_glob_ and E_loc_), controlling for age; 2) To examine whether the changes of network topological properties in liars were associated with their lying behaviors, we conducted a linear regression with the changes of network properties as the predicted variable. Age in years (continuous variable) was entered on the first step, followed by the frequency of lying and the first lie trial on the second step (stepwise).

## Results

### Behavioral results

Preliminary results revealed no significant effect for gender. Thus, the data for this factor were collapsed for all subsequent analyses.

Approximately 68% of children (40 out of 59) lied at least once during the guessing game. Among the children who lied (*n* = 40), the mean first lie trial (FLT) was 11.45^th^ (ranging from 1^st^ to 35^th^, *SD* = 11.10^th^).

The correlational analyses were conducted to examine the relations between age and each of the three measures of children’s dishonest behavior: Decision to lie (i.e., whether they lie or not), frequency of lying, and the FLT (the first lie trial). The results indicated that with increased age, the children begin to lie later and lie more (*r*(40) = 0.370, *p* = 0.019; *r*(40) = 0.322, *p* = 0.043). However, there was no significant relation between the decision to lie (i.e., whether they lied at least once or not) and age. In other words, children lied at least once regardless of age, but with increased age they became more reluctant to lie, consistent with the existing findings^4,32,58^.

## Small-world network analysis results

### The differences in functional connectivity between the honest and dishonest states for liars

To examine whether dishonesty would lead to changes in the network properties, we compared the differences in functional connectivity between the honest state and the dishonest state in liars as indexed by the network topological properties. We divided the time course data of liars into two states (the honest state vs. the dishonest state) using their first lie as the cut-off point.

We conducted the small-world analyses and found that the normalized clustering coefficient (γ) > 1, and normalized characteristic path lengths (λ) ≈ 1. Thus, both the honest and dishonest states had typical features of small-world topology.

**Figure 3:**
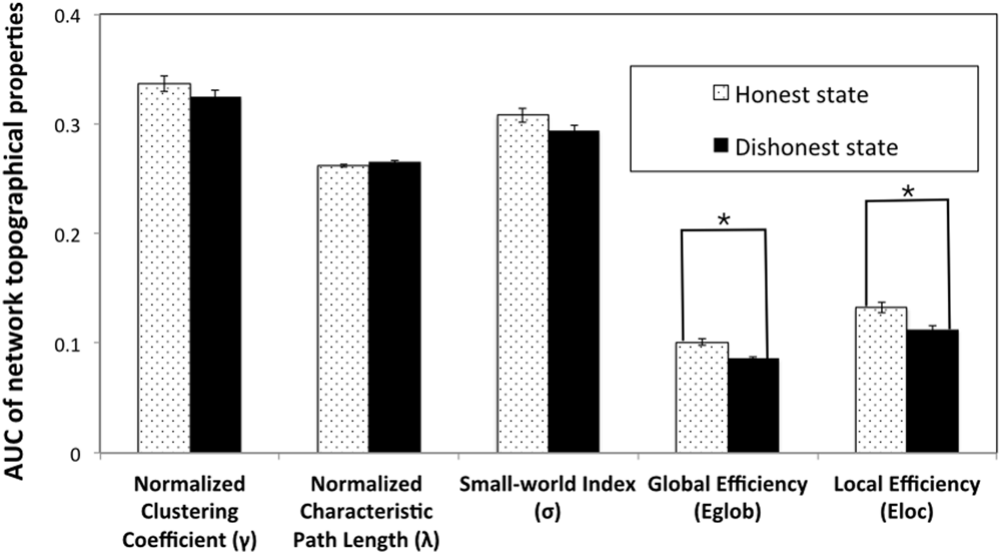
The network properties of the honest state and dishonest state. For global efficiency, the results showed that the main effects of states were significant, *F* (1,38) = 8.26, *p* = 0.007, *η* ^2^ = 0.18. The global efficiency of the dishonest state was significantly less than those of the honest state in liars, suggesting children’s network global efficiency was disrupted after they told the first lie.

**Figure 4:**
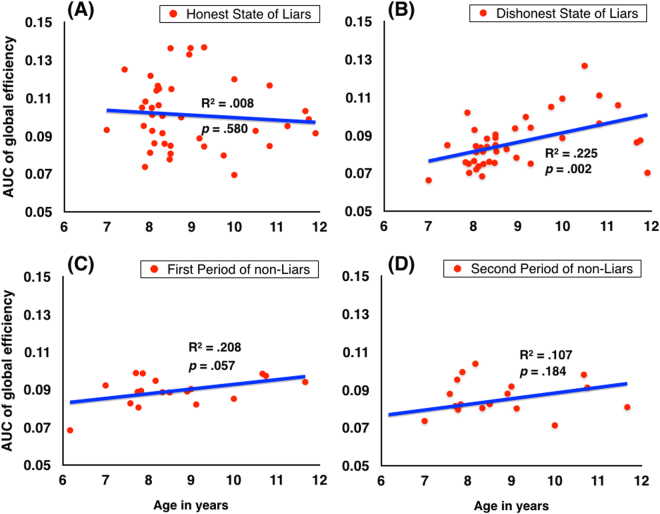
(**A**) The AUC changes of the global efficiency of the honest state in liars as a function of age. (**B**) The AUC of the global efficiency of the dishonest state in liars increased significantly with age. (**C**) The AUC changes of the global efficiency of the first period in non-liars with age. (**D**) The AUC changes of the global efficiency of the second period in non-liars with age.

This main effect of states was modified by a significant States × Age interaction, *F* (1,38) = 5.24, *p* = 0.028, *η* ^2^ = 0.12 (Fig. 3). To determine this significant interaction, Pearson correlations were calculated between the children’s age in years and the global efficiency score for the honest and dishonest state, separately. The age in years was not significant with the global efficiency score of the honest state, *r*(40) = −0.09, *p* = 0.580. Thus, before children told the first lie, the global efficiency of the honest state network was the same regardless of age (Fig. 4A). However, the age in years was significantly correlated with the global efficiency scores of the dishonest state, *r*(40) = 0.47, *p* = 0.002. The global efficiency of younger children’s dishonest state network was significantly lower than that of older children’s (Fig. 4B). Thus, telling the first lie disrupted the younger children’s global efficiency more than that of the older children.

To further illustrate this significant interaction, we subtracted the global efficiency scores of the liars in the dishonest state from those in the honest state. A one-sample t-test revealed that the reduction in global efficiency from before to after the first lie was significantly greater than zero (*t*(39) = −4.19, *p* < 0.001, *Cohen’s d* = 1.34). The Pearson correlation coefficient between the reduction in global efficiency and children’s age in years was also significant (*r*(40) = 0.35, *p* = 0.028), suggesting that with increased age, the reduction in global efficiency after the first lie became increasingly smaller. In other words, lying engendered less network global efficiency disruption as children became older.

For local efficiency, only the main effect of states was significant, *F*(1,38) = 6.07, *p* = 0.018, *η* ^2^ = 0.14: the local efficiency of the dishonest state was significantly less than that of the honest state (Fig. 3). Thus, after children told their first lie, their network local efficiency decreased significantly, again supporting the network disruption hypothesis.

There were no other significant results regarding normalized characteristic path length (λ), normalized clustering coefficient (γ), and network small-worldness (σ).

### The differences between the first and second periods of non-liars

To ascertain whether the liars’ changes in network properties before and after their first lie was not simply due to time-dependent changes such as fatigue. We examined whether non-liars also showed changes in network topological properties in the first and second periods of the experiment similar to those of the liars. We divided the time course data of the non-liars into two periods as described above to compare the network topological properties between the first and second periods in non-liars.

We conducted the functional network analyses. The results of the neural networks for the first and second periods showed that the normalized clustering coefficient (γ) > 1, and normalized characteristic path length (λ) ≈ 1. Thus, both networks had typical features of small-world topology.

We found no significant main effects of periods or age nor their interaction in normalized characteristic path length (λ), normalized clustering coefficient (γ), network small-worldness (σ), global efficiency (E_glob_), or local efficiency (E_loc_) in non-liars. The results suggested that the network topological properties did not change from the first period to the second period in non-liars.

### Comparisons between liars and non-liars

To examine whether the functional connectivity in the liars and non-liars was fundamentally different, we extracted network properties from the whole individual time course data of liars and non-liars, and compared the differences of the network topological properties between the two groups.

The results of these two networks showed that the normalized clustering coefficient (γ) > 1, and normalized characteristic path length (λ)≈1. Thus, both networks had typical features of small-world topology.

We found no significant differences between the liars and non-liars in terms of the normalized characteristic path length (λ), normalized clustering coefficient (γ), network small-worldness (σ), global efficiency (E_glob_), and local efficiency (E_loc_). Only the main effect of age for local efficiency (E_loc_) was significant, *F* (1,55) = 5.45, *p* = 0.023, *η* ^2^ = 0.09, suggesting that, the overall local efficiency (E_loc_) unsurprisingly increased with age. Thus, these results indicated that there were no fundamental differences in network topological properties between the liars and non-liars.

To further examine whether the increase in overall local efficiency with age was specific to deception or merely reflecting some general age differences, we conducted a linear regression with the overall local efficiency as the predicted variable. Age in years (continuous variable) was entered on the first step, followed by the first lie trial and frequency of lying on the second step (stepwise). The results showed that the first model with age was significant, *ΔF*(1, 39) = 4.90, *p* = 0.033, *ΔR* ^2^ = 0.114, suggesting that age was significantly related to the overall local efficiency. Neither the first lie trial nor frequency of lying entered into the second model, suggesting that neither the first lie trial nor the frequency of lying was significantly related to overall local efficiency. The results showed that the overall local efficiency was only related with age, not specific to deception.

### Correlations between behavioral and network measures in the liars

To examine whether the liars’ overall network topological properties were associated with their lying behaviors, we conducted partial correlation analyses between the frequency of lying/FLT (the first lie trial) and the AUCs of each network topological property (γ,λ,σ, E_glob_ and E_loc_), controlling for age. We did not find any significant partial correlations.

In addition, to examine whether the changes in neural networks were associated with children’s lying behaviors, we conducted linear regression analyses with the changes of network properties as the predicted variable. Age in years (continuous variable) was entered on the first step, followed by the first lie trial and frequency of lying on the second step (stepwise). The results showed that, for the changes of global efficiency, the first model with age was significant, *ΔF*(1, 39) = 5.24, *p* = 0.028, *ΔR* ^2^ = 0.12, suggesting that age was significantly related to the changes of network properties after the first lie. In addition, only the first lie trial, not frequency of lying, was significant, *ΔF*(3, 39) = 125.61, p < 0.001, *ΔR* ^2^ = 0.68, suggesting that the first lie trial, not the frequency of lying, was significantly related to the changes of network properties. When examining which specific scores significantly contributed above and beyond all other common contributions in the model, only the first lie trial was found to be significant (*β* = −0.89, *t* = −11.21, *p* < 0.001, part correlation = −0.82). Specifically, for those children who lied, the FLT (first lie trial) was significantly correlated with the changes of the network properties: the earlier participants lied, the more drastic decreases in global efficiency.

For the changes in the local efficiency, the first model with age was not significant, Δ*F*(1, 39) = 3.65 p = 0.06, ΔR^2^ = 0.12, suggesting that age was not significantly related to the changes of local efficiency after the first lie. Only the first lie trial, not frequency of lying, entered into the second model. In addition, the second model with first lie trial was significant, *ΔF*(3, 39) = 35.95, *p* < 0.001, *ΔR* ^2^ = 0.45, suggesting that the first lie trial, not the frequency of lying, was significantly related to the changes of local efficiency after the first lie. When examining which specific scores significantly contributed above and beyond all other common contributions in the model, only the first lie trial was significant (*β* = −0.72, *t* = −6.00, *p* < 0.001, part correlation = −0.67). Specifically, for those children who lied, the FLT (first lie trial) was significantly correlated with the changes of the local efficiency: the earlier participants lied, the more drastic decreases in local efficiency.

## Discussion

The present study examined the neural functional connectivity underlying children’s spontaneous honest and dishonest acts. More specifically, we used the graph theory to measure changes in the network topological properties when children tell lies as opposed to when they tell the truth. We tested whether dishonest behavior would result in “network enhancement” or “network disruption”, and whether this effect, if any existed, would change with age.

We found for the first time that dishonesty negatively affected children’s neural functional connectivity. After they told the first lie, children’s global and local efficiencies decreased significantly. This finding suggested that dishonesty disrupts children’s brain functional organization, supporting the network disruption hypothesis. We further found that the significant reduction of the global efficiency due to dishonesty was affected by age: with increased age, the reduction of the global efficiency became significantly smaller. In other words, the global efficiency of the younger children’s neural network was more susceptible to disruption due to dishonesty than older children. In contrast, the disruption of the local efficiency of the neural network was unaffected by age: regardless of age, the local efficiency decreased significantly after they told the first lie relative to that before they told the first lie.

One could argue that the above findings could be due to time-dependent factors such as fatigue because the dishonest state always followed the honest state. However, when we divided the data of the non-liars into two comparable periods we failed to find significant differences of network properties between the first and second periods. Further, we also found that there were no overall significant differences in the overall network properties between the liars and non-liars, suggesting that the functional neural networks of the liars and non-liars were not fundamentally different from each other in terms of network topological properties. Rather, the liars at some point during the experiment made the decision to lie, whereas the non-liars decided not to lie at all. As the result of their decisions to lie, the liars’ functional connectivity was affected negatively in terms of reductions in both global and local efficiencies.

Why would dishonesty lead to the reduction in network efficiency? According to a recent cognitive theory of deception^25^, there are four cognitive processes in generating deception typically occurring in the following order: an activation component, a decision component, a construction component, and an action component. An activation component refers to the activation of truthful information in semantic or episodic memory, a decision component refers to the intentional decision to lie, the construction component refers to the generation or expression of a plausible alternative to the truth, and an activation component refers to the liar’s delivery of lies to targets. Lying thus involves all the four cognitive components. It is well established that these components engage all aspects of the executive function system such as attention, working memory, inhibition, cognitive switching, and planning^19,58^. In contrast, truth-telling should involve only the first component. Thus, the dishonest act uses more cognitive-neural resources than the honest state^59,60^. Though there was no direct evidence to show that the depletion of cognitive resources will cause the reduction of network efficiency, previous studies have found that cognitive decline or lower intelligence was associated with the loss of network efficiency^34,61–63^. Another study showed that the cognitive training could lead to the enhancement in network efficiency^35^. Taken together, these evidences support the idea that the depletion of cognitive resources would decrease the optimal organization of network, and thus lead to a reduction in network efficiency. According to this “network disruption” hypothesis, in the present study, children’s functional networks were perturbed by their lying and thus decreased significantly in global efficiency and local efficiency.

Regarding the age effect, we found that after telling their first lie, the global efficiency of children’s neural network decreased overall, but the decrease in global efficiency was more severe for younger children than older children. In other words, younger children’s neural network global efficiency was more susceptible to lying than that of the older children. As suggested by several previous studies^64,65^, the global efficiency reflects the cognitive processing capacity of a particular task-related neural network. With increased age, children’s cognitive capacity is known to increase significantly^32,66–68^. Thus, it is possible that due to their increased cognitive capacity, lying did not tax the older children’s cognitive control system as much as it did the younger ones’. As a result, the older children’s global efficiency was less affected by lying than that of the younger ones’.

It should be noted that in contrast to the significant findings concerning global efficiency (E_glob_) and local efficiency (E_loc_) due to the dishonesty, we did not obtain any significant lying-related results regarding normalized characteristic path length (λ), normalized clustering coefficient (γ), or network small-worldness (σ). The reasons for these null results might be that normalized characteristic path length (λ) and normalized clustering coefficient (γ), in contrast to global efficiency (E_glob_) and local efficiency (E_loc_) are measuring different aspects of the topographic properties of the functional neural network based on different assumptions. Lotara and Marchiori (2001) pointed out that the global efficiency (E_glob_) is the efficiency of a parallel system that assumes all the nodes in the network concurrently to exchange packets of information, whereas normalized characteristic path length (λ) measures the efficiency of a sequential system that assumes only one packet of information at a time to go along the network. They suggested that global efficiency (E_glob_) and local efficiency (E_loc_) are the more sensitive measures of the weighted networks than the unweighted networks. One of the reasons is that if a certain node has unusual connections with other nodes, the existence of this node and its connections will have undue influences on the normalized characteristic path length (λ) measure. However, its influences on the global efficiency (E_glob_) are limited. In such cases, the global efficiency measure is a more valid index of this network to measure the efficiency in transporting information. The same is true for the difference between normalized clustering coefficient (γ) and local efficiency (E_loc_) measures^52^.

The present results found that global efficiency and local efficiency decreased significantly due to lying among the liars. Considering that most of the existing studies have exclusively focused on identifying the focal brain areas related to dishonesty^5–7,69,70^, our results suggest that studying the network efficiency changes may be another viable approach to studying the neural mechanisms underlying deception. Also, the network efficiency changes may be used for deception detection^16–18^. In addition, although the present study only focused on children, it is possible that our findings could be extended to adults because several studies with adults showed that functional network topological properties change when adults are on task versus when they are off task^63,71,72^. It is thus possible that adults’ network topological properties may also change after they lie as long as the lying task is sufficiently challenging. Furthermore, in the present study, we only focused on the frontal-parietal networks, which are believed to be the core network for cognitive control. Future studies need to investigate not only the cognitive control networks but also those involved in theory of mind reasoning and reward evaluation, because the existing neural imaging studies have identified theory of mind and reward evaluation to play important roles in deception^5,73,74^.

## Conclusion

The present study examined the changes in neural network topographical properties associated with children’s honest and dishonest behavior based on the graph-theory. To our best knowledge, this is the first study to take a complex network approach to analyze neural response data when children are spontaneously engaged in honest or dishonest acts. We found the brain functional organization is disrupted after children tell a lie, supporting the network disruption hypothesis. Further, lying disrupts the local efficiency of children’s functional neural network equally regardless of their age. However, lying disrupts younger children’s global efficiency more than that of older children, suggesting that with age, children’s frontal-parietal neural network becomes increasingly capable of meeting the cognitive demands imposed by the task of lying. Taken together, the findings of this study suggest that lying disrupts the efficiency of children’s cortical network functioning. Further, they suggest that the graph theory based network analysis is a viable approach to study the neural development of deception.
